# Efferocytosis potentiates the expression of arachidonate 15-lipoxygenase (ALOX15) in alternatively activated human macrophages through LXR activation

**DOI:** 10.1038/s41418-020-00652-4

**Published:** 2020-11-11

**Authors:** Ryan G. Snodgrass, Yvonne Benatzy, Tobias Schmid, Dmitry Namgaladze, Malwina Mainka, Nils Helge Schebb, Dieter Lütjohann, Bernhard Brüne

**Affiliations:** 1 Faculty of Medicine, Institute of Biochemistry I, Goethe-University Frankfurt, Frankfurt, Germany; 2 Chair of Food Chemistry, Faculty of Mathematics and Natural Sciences, University of Wuppertal, Wuppertal, Germany; 3 Institute for Clinical Chemistry and Clinical Pharmacology, University of Bonn, Bonn, Germany

**Keywords:** Sterols, Cell death and immune response, Chronic inflammation

## Abstract

Macrophages acquire anti-inflammatory and proresolving functions to facilitate resolution of inflammation and promote tissue repair. While alternatively activated macrophages (AAMs), also referred to as M2 macrophages, polarized by type 2 (Th2) cytokines IL-4 or IL-13 contribute to the suppression of inflammatory responses and play a pivotal role in wound healing, contemporaneous exposure to apoptotic cells (ACs) potentiates the expression of anti-inflammatory and tissue repair genes. Given that liver X receptors (LXRs), which coordinate sterol metabolism and immune cell function, play an essential role in the clearance of ACs, we investigated whether LXR activation following engulfment of ACs selectively potentiates the expression of Th2 cytokine-dependent genes in primary human AAMs. We show that AC uptake simultaneously upregulates LXR-dependent, but suppresses SREBP-2-dependent gene expression in macrophages, which are both prevented by inhibiting Niemann–Pick C1 (NPC1)-mediated sterol transport from lysosomes. Concurrently, macrophages accumulate sterol biosynthetic intermediates desmosterol, lathosterol, lanosterol, and dihydrolanosterol but not cholesterol-derived oxysterols. Using global transcriptome analysis, we identify anti-inflammatory and proresolving genes including interleukin-1 receptor antagonist (IL1RN) and arachidonate 15-lipoxygenase (ALOX15) whose expression are selectively potentiated in macrophages upon concomitant exposure to ACs or LXR agonist T0901317 (T09) and Th2 cytokines. We show priming macrophages via LXR activation enhances the cellular capacity to synthesize inflammation-suppressing specialized proresolving mediator (SPM) precursors 15-HETE and 17-HDHA as well as resolvin D5. Silencing LXRα and LXRβ in macrophages attenuates the potentiation of ALOX15 expression by concomitant stimulation of ACs or T09 and IL-13. Collectively, we identify a previously unrecognized mechanism of regulation whereby LXR integrates AC uptake to selectively shape Th2-dependent gene expression in AAMs.

## Introduction

Due to their inherent plasticity, macrophages play critical roles in acute inflammatory responses through both directing inflammation and tissue destruction, as well as promoting tissue repair and healing [1]. During the induction phase of inflammation pro-inflammatory macrophages release a variety of lipid and protein mediators to amplify the immune response [2]. Following neutralization of the initial insult, inflammation must be curtailed to prevent persistent-chronic inflammation and allow for the repair of damaged tissue. This multistep process, referred to as the resolution phase of inflammation, involves cessation of immune cell infiltration, counter-regulation of chemokines and cytokines, clearance of apoptotic cells (ACs), and the initiation of tissue healing that culminates in a return to tissue homeostasis [2].

How macrophages acquire anti-inflammatory and proresolving functions have been under intense investigation. Of the numerous signals that instruct macrophage polarization for the final stages of wound healing, Th2 cytokines interleukin (IL)-4 and IL-13 as well as ACs are major drivers of the process [2, 3]. Macrophages activated by Th2 cytokines are referred to as alternatively activated macrophages (AAMs), or M2 macrophages [4, 5]. Th2 cytokine-induced polarization is dominated by activation of the signal transducer and activator of transcription 6 (STAT6) transcription factor [6] and is characterized by increased expression of chemokines, nucleotide, and scavenger receptors, as well as lipid metabolism signaling genes which facilitate the biosynthesis of specialized proresolving mediators (SPMs) that promote resolution and tissue regeneration [4, 7, 8].

The collective process of recognizing, engulfing, and digesting ACs, referred to as efferocytosis, suppresses inflammatory gene expression and prevents immune responses against self-antigens [9, 10]. Although recognition and engulfment of dying cells are well studied [11], transcriptional pathways that integrate AC clearance and mediate the immunosuppressive effects are less characterized [12]. Many transcriptional changes induced by efferocytosis are mediated by members of the nuclear hormone receptor superfamily including peroxisome proliferator activated receptors (PPARs), retinoid X receptors (RXRs), retinoic acid receptors (RARs), and liver X receptors (LXRs) [12]. Macrophage-specific deletion of PPARƴ or RXRα impairs AC engulfment leading to glomerular damage and elevated autoantibody production [13]. Macrophages deficient in LXRs (LXRα and LXRβ), which are critical for linking sterol metabolism and immune cell function [9, 14], are unable to clear ACs, while LXR-deficient mice accumulate dead cells and develop systemic autoimmune disease [9]. LXR activity is induced under conditions of cholesterol excess, however rather than sense cholesterol directly, LXRs are instead positively regulated by oxysterols and sterol intermediates of the cholesterol biosynthetic pathway [15]. LXRs heterodimerize with RXR and bind DNA sequences termed LXR response elements (LXREs) in the transcriptional regulatory regions of their target genes to facilitate cholesterol efflux [14]. In addition to reducing the sterol burden, LXR indirectly promotes efferocytosis through upregulating its own expression in an autoregulatory manner [16], as well as expression of RARα [17] and MERTK [9].

ACs and Th2 cytokines act in concert to enhance the capacity of macrophages to induce anti-inflammatory and tissue repair phenotypes [3]. Although stimulation with IL-4 or IL-13 upregulates a broad set of genes, concomitant exposure with ACs selectively potentiated the expression of anti-inflammatory and tissue repair genes including *Retnla*, *Chil3*, and *Ear2*, but not the expression of canonical Th2 cytokine-dependent genes in murine bone marrow-derived macrophages [3].

Given that LXRs coordinate sterol metabolism and immune cell function, we investigated whether LXR activation following engulfment of ACs selectively potentiates the expression of Th2 cytokine-dependent genes in primary human AAMs. We show that efferocytic macrophages accumulate sterol intermediates and exhibit enhanced LXR-, but reduced SREBP-2-dependent gene expression. Using global transcription analysis, we identify arachidonate 15-lipoxygenase (ALOX15) and IL-1 receptor antagonist (IL1RN) as genes whose expression is selectively potentiated in macrophages upon concomitant exposure to ACs and Th2 cytokines. Furthermore, priming macrophages through LXR activation does not potentiate common Th2 cytokine-dependent genes associated with the AAM phenotype but selectively enhances ALOX15 expression and the capacity to synthesize inflammation-suppressing SPM precursors and SPMs. Collectively, we identify a previously unrecognized mechanism of regulation whereby efferocytosis potentiates ALOX15 expression in AAMs through LXR activation.

## Materials and methods

### Materials

T0901317 (catalog no. 2373) was purchased from Tocris Bioscience (Wiesbaden-Nordenstadt, Germany), U-18666A (catalog no. BML-S200) from Enzo Life Sciences (Lausen, Switzerland), staurosporine (catalog no. S-9300) from LC Laboratories (Woburn, MA, USA), triparanol (catalog no. T5200) and calcium ionophore A23187 (catalog no. C7522) from Merck (Darmstadt, Germany), desmosterol (catalog no. 14943), peroxide-free arachidonic acid (AA) (catalog no. 17948), eicosapentaenoic acid (EPA) (catalog no. 17949), and docosahexaenoic acid (DHA) (catalog no. 17950) from Cayman Chemical (Ann Arbor, MI, USA), and recombinant human IL-4 (catalog no. 200-04) and IL-13 (catalog no. 200-13) from PeproTech (Hamburg, Germany). Primers were purchased from Biomers GmbH (Ulm, Germany) and their sequences are available on request. All chemicals were of the highest grade of purity and commercially available.

### Primary human macrophage generation

Human monocytes were isolated from commercially obtained buffy coats from anonymous donors (DRK-Blutspendedienst Baden-Württemberg-Hessen, Institut für Transfusionsmedizin und Immunhämatologie, Frankfurt, Germany) using Ficoll density centrifugation. Peripheral blood mononuclear cells were washed twice with PBS containing 2 mM EDTA and subsequently incubated for 1 h at 37 °C in RPMI 1640 medium supplemented with penicillin (100 U/ml) and streptomycin (100 μg/ml) to allow their adherence to culture dishes (Sarstedt, Nümbrecht, Germany). Nonadherent cells were removed. Monocytes were then differentiated into naive macrophages with RPMI 1640 medium containing 5% AB-positive human serum (DRK-Blutspendedienst Baden-Württemberg-Hessen, Frankfurt, Germany) for 7 days. Naive macrophages were polarized to AAMs with IL-4 (5 ng/ml) or IL-13 (10 ng/ml), and primed with T0901317 (1 µM) unless indicated otherwise.

### AC preparation

Human Jurkat T cells (catalog no. ACC 282, DSMZ, Braunschweig, Germany) were treated with 0.5 µM staurosporine in serum-free RPMI 1640 for 3 h, washed extensively with RPMI 1640 medium containing human serum, then cocultured with human macrophages (1:10 macrophage to AC ratio) for 3 h. Cell death was routinely controlled and quantified by staining with annexin V/propidium iodide (Immunotools, Friesoythe, Germany) by FACS analysis (LSR Fortessa, BD Life Sciences, Heidelberg, Germany).

### siRNA transfections

Control siRNA and siRNAs targeting human LXRα and LXRβ (siRNA; siGENOME human SMARTpool, Thermo Fisher Scientific, Karlsruhe, Germany) were transfected into primary human macrophages at a final concentration of 50 nM using HiPerFect transfection reagent (Qiagen, Hilden, Germany) according to the manufacturer’s recommendations. Each knockdown was routinely confirmed by qRT-PCR for each experiment.

### RNA extraction and quantitative real-time PCR

Total RNA from macrophages was isolated using PeqGOLD RNAPure reagent (PeqLab Biotechnologie, Erlangen, Germany) and quantified using the NanoDrop spectrophotometer (NanoDrop, Wilmington, DE, USA). Total RNA (1 μg) was transcribed with the Maxima First Strand cDNA Synthesis Kit (Thermo Fisher Scientific). Quantitative real-time PCR was performed using iQ SYBR green Supermix (Bio-Rad Laboratories, Munich, Germany) and the Bio-Rad CFX96 system. Primer sequences for quantitative PCR can be obtained upon request. β2 microglobulin was used as an endogenous control for human macrophages.

### RNA-sequencing library preparation

Total RNA was isolated from macrophages using RNeasy Mini Kit (Qiagen), purified by precipitation, then quality was assessed using an RNA 6000 Nano Chip with an Agilent 2100 Bioanalyzer (Agilent Technologies, Waldbronn, Germany). Quantification was carried out using Qubit HS RNA Assay Kit and Qubit 4 fluorometer (Thermo Fisher Scientific). Library preparation of 500 ng RNA was performed using the Lexogen QuantSeq 3′ mRNA-Seq Library Prep Kit FWD for Illumina (Lexogen GmbH, Vienna, Austria). Afterward, cDNA libraries were controlled for quantity and quality by Qubit 4 fluorometer and Agilent High Sensitivity DNA Chip using the Agilent 2100 Bioanalyzer. The average size of the libraries was calculated within 150–1000 bp and a 1 nM pool of all libraries was denatured, diluted, and combined with a PhiX library control. Subsequently, libraries were sequenced (single-end, 75 cycles) using the High Output Kit v2 on an Illumina NextSeq 500 sequencer (Illumina, San Diego, CA, USA). Sequencing data have been deposited under the GEO accession number GSE158022.

### RNA-sequencing data analysis

Illumina-generated FASTQ files were processed using the Lexogen QuantSeq 3′ mRNA-Seq integrated data analysis pipeline on the BlueBee online platform (BlueBee Holding BV, Rijswijk, The Netherlands). Within the integrated data analysis pipeline, reads were trimmed using bbduk v35.92, were mapped to the hg38 human genome assembly using STAR Aligner v2.5.2a with modified ENCODE settings, and gene-specific read counts were determined by HTSeq-count v0.6.0. The read count files were used as input for edgeR (Galaxy Version 3.24.1) [18] to calculate differentially expressed genes. To identify upregulated genes by IL-13, IL-4, and T0901317 we filtered the differentially expressed gene lists from edgeR by requiring the minimum count-per-million (CPM) threshold be greater than 1, the fold change in expression be greater than or equal to 1.5, and the quasi-likelihood *F*-test value be equal to or greater than 10.

To identify Th2 cytokine-dependent genes potentiated by LXR priming with T0901317, we used the unfiltered differentially expressed gene lists from edgeR comparing stimulations with IL-13 and IL-4 alone to those of T0901317 + IL-13 and T0901317 + IL-4, respectively. We filtered the two lists by requiring the fold change in expression following stimulation with T0901317 alone be less than 1.5 and the fold change in expression following stimulation with IL-13 or IL-4 alone be greater than 0. Next, we filtered the gene lists by including only those genes which demonstrated a fold change in expression greater or equal to 1.5 with a CPM greater than 5 on both lists.

### Immunoblots

Macrophages lysates were resolved on polyacrylamide gels followed by transfer onto nitrocellulose membranes. Membranes were blocked with 5% milk/100 mM Tris–HCl, 150 mM sodium chloride, 0.01% (v/v) Tween 20 (TTBS) followed by incubation with antibodies against ALOX15 (catalog no. ab119774, Abcam, Cambridge, UK), DHCR24 (catalog no. 10471-1-AP, Proteintech, St. Leon-Rot, Germany), or nucleolin (catalog no. sc-55486, Santa Cruz Biotechnology, Heidelberg, Germany). For protein detection, the membrane was incubated with IRDye secondary antibodies (LI-COR Biosciences, Bad Homburg, Germany) in 5% BSA/TTBS. Proteins were visualized and when applicable densitometrically analyzed with the Odyssey infrared imaging system (LI-COR Biosciences).

### Measurement of cellular cholesterol, noncholesterol sterol, and oxysterol content

Cholesterol, noncholesterol sterol, and oxysterol content was analyzed in macrophages and Jurkat cells by gas chromatography (GC). Cell pellets were spun in a speedvac concentrator (12 mbar; Savant AES 1000) and weighed. Cholesterol, noncholesterol sterols, and oxysterols were extracted using chloroform. After alkaline hydrolysis, the concentrations of cholesterol precursors were measured with GC-mass spectrometry-selected ion monitoring [19]. The trimethylsilyethers of the sterols were separated on a DB-XLB (30 m length × 0.25 mm internal diameter, 0.25 μm film) column (Agilent Technologies, Waldbronn, Germany) using the 6890N Network GC system (Agilent Technologies). Epicoprostanol (Steraloids, Newport, RI, USA) was used as an internal standard, to quantify the noncholesterol sterols (Medical Isotopes, Pelham, NH, USA) on a 5973 Network MSD (Agilent Technologies). Total cholesterol was measured by GC-flame ionization detection on an HP 6890 GC system (Hewlett Packard, Waldbronn, Germany), equipped with a DB-XLB (30 m length × 0.25 mm internal diameter, 0.25 μm film) column (Agilent Technologies) using 5a-cholestane (Steraloids) as internal standard [20].

### Lipid mediator formation and analysis

To stimulate lipid mediator formation, macrophages were incubated at 5 × 10^6^ cells/ml of PBS containing glucose, Ca^2+^, Mg^2+^, AA (1.5 µM), EPA (1.5 µM), DHA (1.5 µM), and calcium ionophore (2.5 µM) at 37 °C. After 30 min, an equal volume of MeOH was added to cell suspensions. Samples were sonicated and snap frozen. Lipid mediator formation was analyzed by means of LC–ESI–MS/MS as described [21, 22]. In brief, cells were sonicated in 1 ml PBS buffer/MeOH (50/50, v/v) and mixed with 10 µl deuterated internal standards (100 nM, containing ^2^H_5_-RvD2, ^2^H_5_-LxA_4_, ^2^H_5_-RvD1, ^2^H_4_-LTB_4_, ^2^H_4_-9,10-DiHOME, ^2^H_8_-15-HETE, ^2^H_8_-12-HETE, ^2^H_8_-5-HETE) and 10 µl antioxidant mixture (0.2 mg/ml BHT, 100 µM indomethacin, 100 µM trans-4-[4-(3-adamantan-1-yl-ureido)-cyclohexyloxy]-benzoic acid (t-AUCB). Following addition of 900 µl MeOH and storage at −80 °C for 30 min precipitated proteins were removed by centrifugation (10 min, 20,000 × *g*, 4 °C). The supernatants were evaporated to <50% MeOH in a vacuum concentrator. Samples were diluted with 2 ml of 0.1 M disodium hydrogen phosphate buffer (adjusted to pH 6 with acetic acid) and extracted by solid phase extraction using Bond Elut Certify II cartridges (200 mg, 3 ml, Agilent Technologies). Oxylipins were eluted using ethyl acetate/*n*-hexane (75/25, v/v) with 1% acetic acid, evaporated to dryness (vacuum concentrator, 30 °C, 1 mbar), and reconstituted in 50 µl MeOH containing 40 nM of each, 1-(1-(ethylsulfonyl)piperidin-4-yl)-3-(4-(trifluoromethoxy)phenyl)urea, 12-(3-adamantan-1-yl-ureido)-dodecanoic acid, 12-oxo-phytodienoic acid, and aleuritic acid, as IS2. The LC–MS analysis of mono- and multiple-hydroxylated polyunsaturated fatty acids (PUFAs) was carried out using a 1290 LC System (Agilent Technologies) with a 5500 QTrap (Sciex, Darmstadt, Germany) as described [21, 22]. Five microliters of fatty acids were injected and the instrument was operated in scheduled selected reaction monitoring. Quantification was based on an external calibration with characterized standards [23] using the above-mentioned internal standards. Limit of detection and quantifications are reported in [21, 22].

### Statistical analysis

Graphical data are presented as means ± SE of at least three independent experiments using human monocyte-derived macrophages derived from different individual donors, unless noted otherwise in the figure legends. Sample size for each experiment was estimated empirically, according to the exploratory experiments and published literatures with similar methodology. Differences were considered significant when *P* < 0.05 (*P < 0.05; **P < 0.01; ***P < 0.001; ns = not significant). Statistical significance was calculated using one-way analysis of variance (ANOVA) with Bonferroni post hoc test, two-tailed Student’s *t* test with significance level set at 0.05, or one sample *t*-test with significance level set at 0.05 using Prism 8 software (GraphPad, La Jolla, CA, USA). Final assembly and preparation of all figures was done using CorelDRAW 2018 (Corel Corporation, Ottawa, Canada).

## Results

### Sterol intermediates modulate LXR- and SREBP-2-dependent gene expression in human efferocytic macrophages

**Fig. 1:**
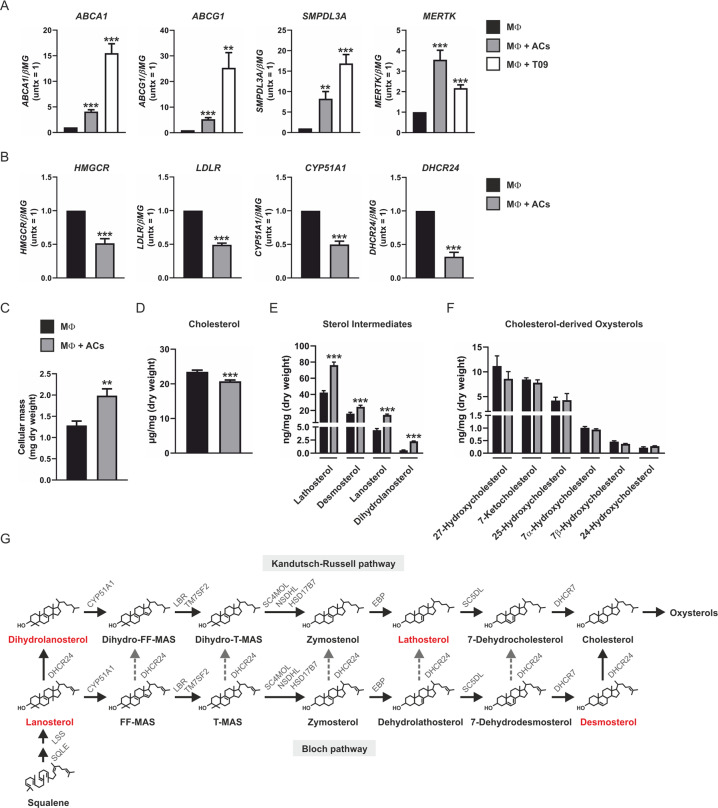
LXR- and SREBP-2-dependent gene expression in efferocytic macrophages. Expression of **a** LXR-dependent genes ABCA1, ABCG1, SMPDL3A, and MERTK in primary human monocyte-derived naive macrophages cocultured with apoptotic Jurkat cells or stimulated with T09 (1 µM) for 3 h and **b** SREBP-2-dependent genes HMGCR, LDLR, CYP51A1, and DHCR24 in naive macrophages cocultured with apoptotic Jurkat cells for 3 h. **c** Cellular mass, **d** total cholesterol, **e** sterol intermediates, and **f** cholesterol-derived oxysterols in naive macrophages and macrophages following 3-h coculture with apoptotic Jurkat cells. **g** Schematic representation of the Bloch and Kandutsch–Russell pathways for the enzymatic conversion of squalene to cholesterol. Potential sites of crossover from Bloch to Kandutsch–Russell pathway are indicated by broken gray arrows. Intermediates increased in efferocytic macrophages are shown in red. Data are presented as mean ± SE from at least four independent experiments. Statistical analysis was performed using one sample *t*-test for **a** and **b** and two-tailed Student’s *t* test for **c**–**f** (**P < 0.01 and ***P < 0.001 vs control macrophages).

Given the role of LXR in clearing ACs [9], we posited that LXR functions as a signaling node coupling engulfment of ACs to enhanced Th2 cytokine-dependent gene expression. To explore this relationship, we examined the extent to which ACs induce LXR-dependent gene expression. For this we used an in vitro model of efferocytosis consisting of human monocyte-derived macrophages cocultured with apoptotic Jurkat cells. As assessed by FACS analysis, ACs were efficiently engulfed by macrophages after 1-h coculture (Supplementary Fig. 1). Furthermore, 3-h coculture with ACs or stimulation with synthetic LXR ligand T0901317 (T09) increased expression of classic LXR-dependent genes ABCA1, ABCG1, SMPDL3A, and MERTK (Fig. 1a). Interestingly, MERTK expression was substantially higher in efferocytic macrophages compared to T09-stimulated cells suggesting additional LXR-independent mechanisms of regulation. Since sterol homeostasis is maintained by reciprocal regulation of LXR and SREBP-2 transcriptional programs, we also measured expression of SREBP-2-dependent genes following exposure to ACs. These results showed HMGCR, LDLR, CYP51A1, and DHCR24 were all coordinately downregulated in efferocytic macrophages (Fig. 1b).

To identify endogenous ligands mediating simultaneous and reciprocal LXR- and SREBP-2-dependent signaling we measured sterols in macrophages following 3-h coculture with ACs using GC-MS. Despite an increase in mass, total cholesterol levels were lower in efferocytic macrophages, as a percentage of dry weight (Fig. 1c, d). In contrast, sterol intermediates lathosterol, desmosterol, lanosterol, and dihydrolanosterol were all significantly increased in efferocytic macrophages (Fig. 1e). No alterations in levels of cholesterol-derived 24-, 25-, 27-, 7α-, 7β-hydroxycholesterol (HC), or 7-ketocholesterol were observed (Fig. 1f). Collectively, our reciprocal LXR- and SREBP-2-dependent gene expression profiles in efferocytic macrophages containing increased levels of sterol intermediates of the Kandutsch–Russell and Bloch pathways are in line with previous reports showing sterol intermediates simultaneously activate LXR and suppress SREBP-2 target genes (Fig. 1g) [15, 24, 25].

### Exogenous and endogenous sterol intermediates reciprocally regulate LXR- and SREBP-2-dependent gene expression

**Fig. 2:**
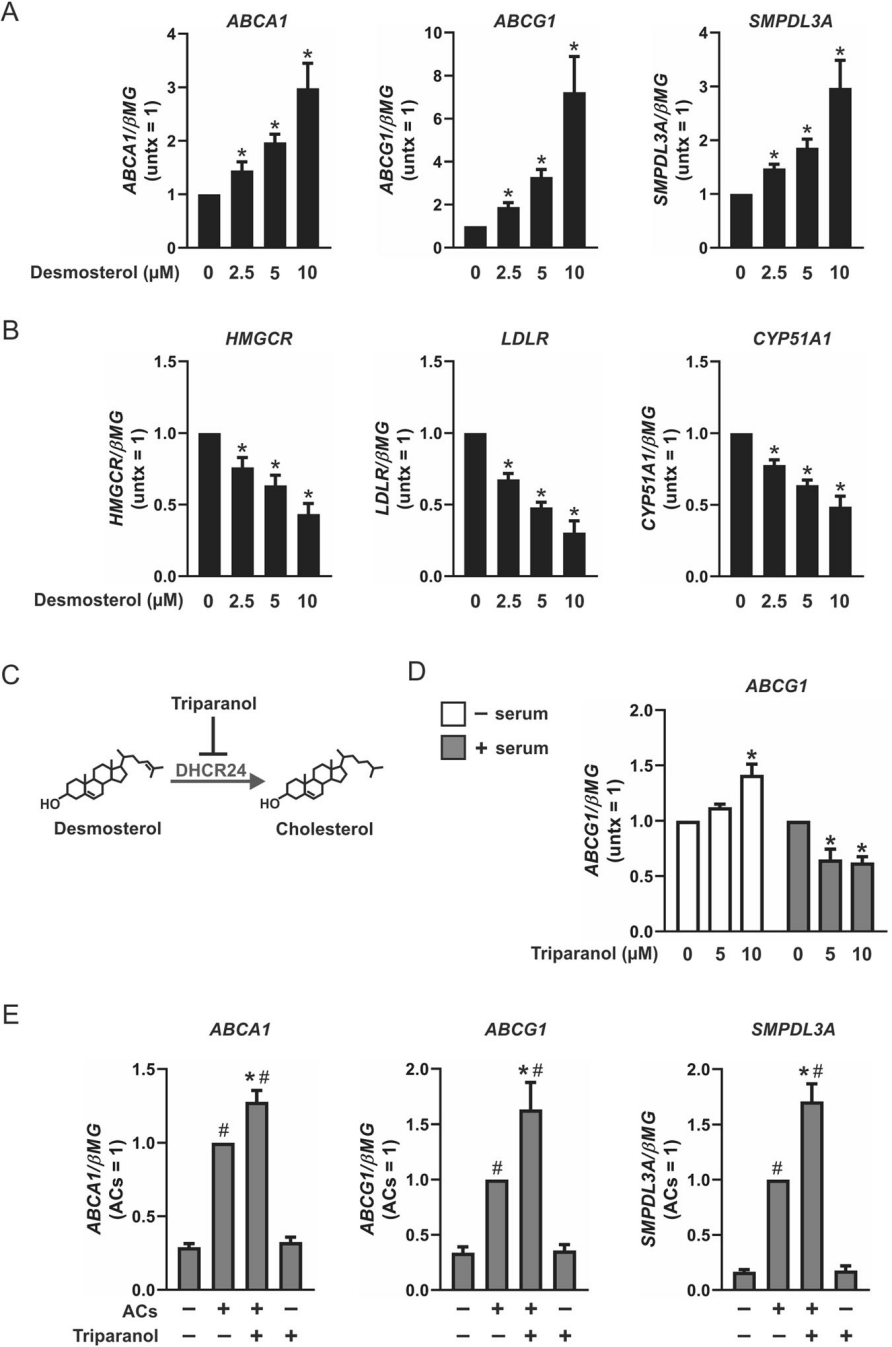
Exogenous and endogenous sterol intermediates reciprocally regulate LXR- and SREBP-2-dependent gene expression. Expression of **a** LXR-dependent genes ABCA1, ABCG1, and SMPDL3A, and **b** SREBP-2-dependent genes HMGCR, LDLR, and CYP51A1 in human monocyte-derived naive macrophages treated with desmosterol (2.5, 5, and 10 µM) for 24 h. **c** Action of triparanol, a pharmacological inhibitor of 24-dehydrocholesterol reductase, which has been shown to increase endogenous levels of desmosterol. **d** Gene expression of ABCG1 in macrophages treated with triparanol (5 and 10 µM) with (inactive cholesterol biosynthesis) and without (active cholesterol biosynthesis) serum for 24 h. **e** Expression of LXR-dependent genes ABCA1, ABCG1, and SMPDL3A in naive macrophages cotreated with triparanol (10 µM) and ACs for 3 h. Data are presented as mean ± SE from at least three independent experiments. Statistical analysis was performed using one sample *t*-test for **a** and **b** (*P < 0.05 vs 0 µM desmosterol), and **d** (*P < 0.05 vs 0 µM triparanol), and one-way ANOVA with Bonferroni post hoc means comparisons for **e** (*P < 0.05 vs ACs; ^#^*P* < 0.05 vs untreated control).

Unlike cholesterol, which suppresses SREBP-2 processing but does not activate LXR, many sterol intermediates coordinately and reciprocally regulate SREBP-2 and LXR activities [15, 24–26]. To determine whether the accumulation of sterol intermediates observed in efferocytic macrophages modulate LXR and SREBP-2 activities, we treated macrophages with desmosterol. As shown in Fig. 2a, desmosterol dose dependently increased expression of LXR target genes ABCA1, ABCG1, and SMPDL3A and simultaneously reduced expression of SREBP-2-dependent genes HMGCR, LDLR, and CYP51A1. To determine whether accumulation of endogenous sterol intermediates recapitulate the effects of ACs with respect to LXR-dependent gene expression we used triparanol (Fig. 2c), a pharmacological inhibitor of 24-dehydrocholesterol reductase (DHCR24) shown to increase endogenous levels of desmosterol, zymosterol, and lathosterol [25, 27]. Consistent with a recent report which inhibited DHCR24 in RAW264.7 cells [28], triparanol increased ABCG1 gene expression in macrophages with active cholesterol biosynthesis (cultured in media free of serum cholesterol), but not in cells without active cholesterol synthesis (cultured in media containing serum cholesterol) (Fig. 2d). Since triparanol causes ABCG1 regulation only in conjunction with active cholesterol biosynthesis, we next determined whether triparanol further enhances the expression of LXR-dependent genes in efferocytic macrophages with accumulated sterol intermediates. Macrophages cotreated with triparanol and ACs showed enhanced expression of ABCA1, ABCG1, and SMPDL3A, compared to cells cocultured with ACs alone (Fig. 2e). These results show the accumulation of sterol intermediates, either through pharmacological means or engulfment of ACs, upregulates LXR-dependent gene expression in macrophages.

### Impact of LXR activation on AAMs

**Fig. 3:**
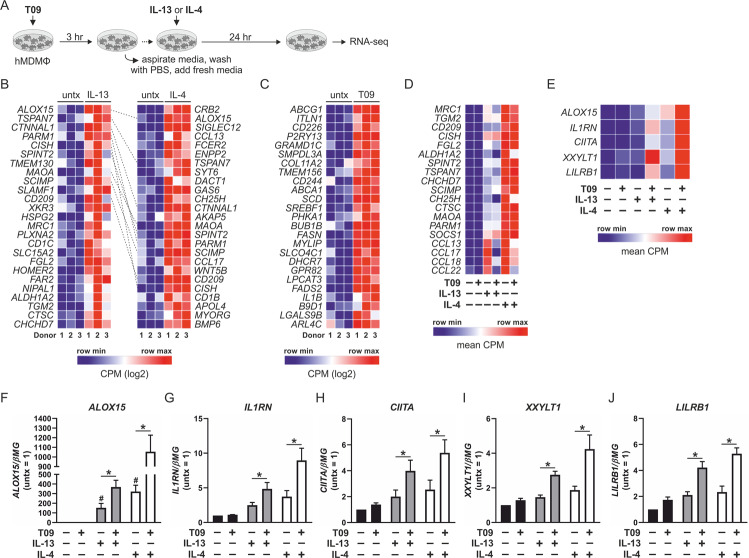
Identification of Th2 cytokine-dependent genes potentiated by LXR. **a** Schematic representation of treatment protocol. Primary human monocyte-derived naive macrophages (hMDMФs) were pulsed with T09 (1 µM) for 3 h. Culture media was aspirated and cells washed with PBS then stimulated with Th2 cytokines IL-4 (5 ng/ml) or IL-13 (10 ng/ml) for 24 h before performing RNA-seq analysis. Heat map displaying 25 strongest upregulated genes as count-per-million (CPM) following **b** 24-h stimulation with IL-13 or IL-4 and **c** 3-h pulse with T09. Dashed gray lines connect corresponding genes. Heat map displaying **d** the impact of T09 on the expression of Th2 cytokine-dependent genes associated with the AAM transcriptional profile as mean CPM and **e** the expression of Th2 cytokine-dependent genes ALOX15, IL1RN, CIITA, XXYLT1, and LILRB1 potentiated by LXR activation as mean CPM. Validation of **f** ALOX15, **g** IL1RN, **h** CIITA, **i** XXYLT1, and **j** LILRB1 gene expression in naive macrophages pulsed with T09 for 3 h followed by stimulation with IL-4 or IL-13 for 24 h by real-time PCR. Data presented in **b**–**e** are from hMDMФs derived from three individual donors. Data presented in **f**–**j** are mean ± SE from at least three independent experiments. Statistical analysis was performed using one-way ANOVA with Bonferroni post hoc means comparisons (*P < 0.05; ^#^*P* < 0.05 vs untreated control).

To test our hypothesis that efferocytosis-induced LXR activation potentiates expression of Th2 cytokine-dependent genes, we performed RNA-seq experiments. To delineate LXR-dependent from LXR-independent signaling, we pulsed macrophages with T09 rather than coculturing with ACs, followed by stimulation with Th2 cytokines (Fig. 3a). Consistent with previously reported transcriptional profiles of human AAMs, stimulation with IL-4 or IL-13 alone upregulated the expression of many analogous genes including ALOX15, MRC1, CD209, MAOA, and TGM2. Heat maps indicate the top 25 most upregulated genes in IL-13- and IL-4-stimulated macrophages (Fig. 3b) [4, 5]. Stimulation with 5 ng/ml IL-4 upregulated the expression of 175 genes more than 1.5-fold (Supplementary Table 1) compared to only 32 genes following treatment with 10 ng/ml IL-13 (Supplementary Table 2). A 3-h pulse with T09 upregulated the expression of 171 genes (Supplementary Table 3) including cholesterol transporters ABCG1 and ABCA1, phospholipid remodeling enzyme LPCAT3, as well as nuclear receptor RARα. Heat map shows the top 25 most upregulated genes in T09-stimulated macrophages (Fig. 3c). Analysis of our differentially expressed gene sets showed many common Th2 cytokine-dependent genes associated with the AAM transcriptional profile including MRC1, TGM2, CD209, ALDH1A2, and MAOA, as well as chemokines CCL13, CCL17, CCL18, and CCL22 were not potentiated by LXR priming, while expression of CHCHD7, SOCS1, and SCIMP showed modest additive effects of LXR activation (Fig. 3d and Supplementary Table 4). To identify unique Th2 cytokine-dependent genes potentiated by LXR, we used differentially expressed gene lists from macrophages pulsed with T09 then stimulated with Th2 cytokine IL-13 or IL-4 compared with cells stimulated with the respective Th2 cytokine alone. After excluding genes upregulated by T09 alone (LXR-responsive genes) and genes downregulated by IL-13 or IL-4 alone, we selected those Th2 cytokine-dependent genes whose expression was potentiated by T09 more than 1.5-fold. Using this criteria, we identified five IL-4- and IL-13-dependent genes whose expression was strongly potentiated by LXR activation; ALOX15, IL1RN, class II major histocompatibility complex transactivator (CIITA), xyloside xylosyltransferase 1 (XXYLT1), and leukocyte immunoglobulin like receptor B1 (LILRB1) (Fig. 3e and Supplementary Table 5). Their expression was confirmed using qPCR (Fig. 3f–j).

### ALOX15 expression is modulated by LXR

**Fig. 4:**
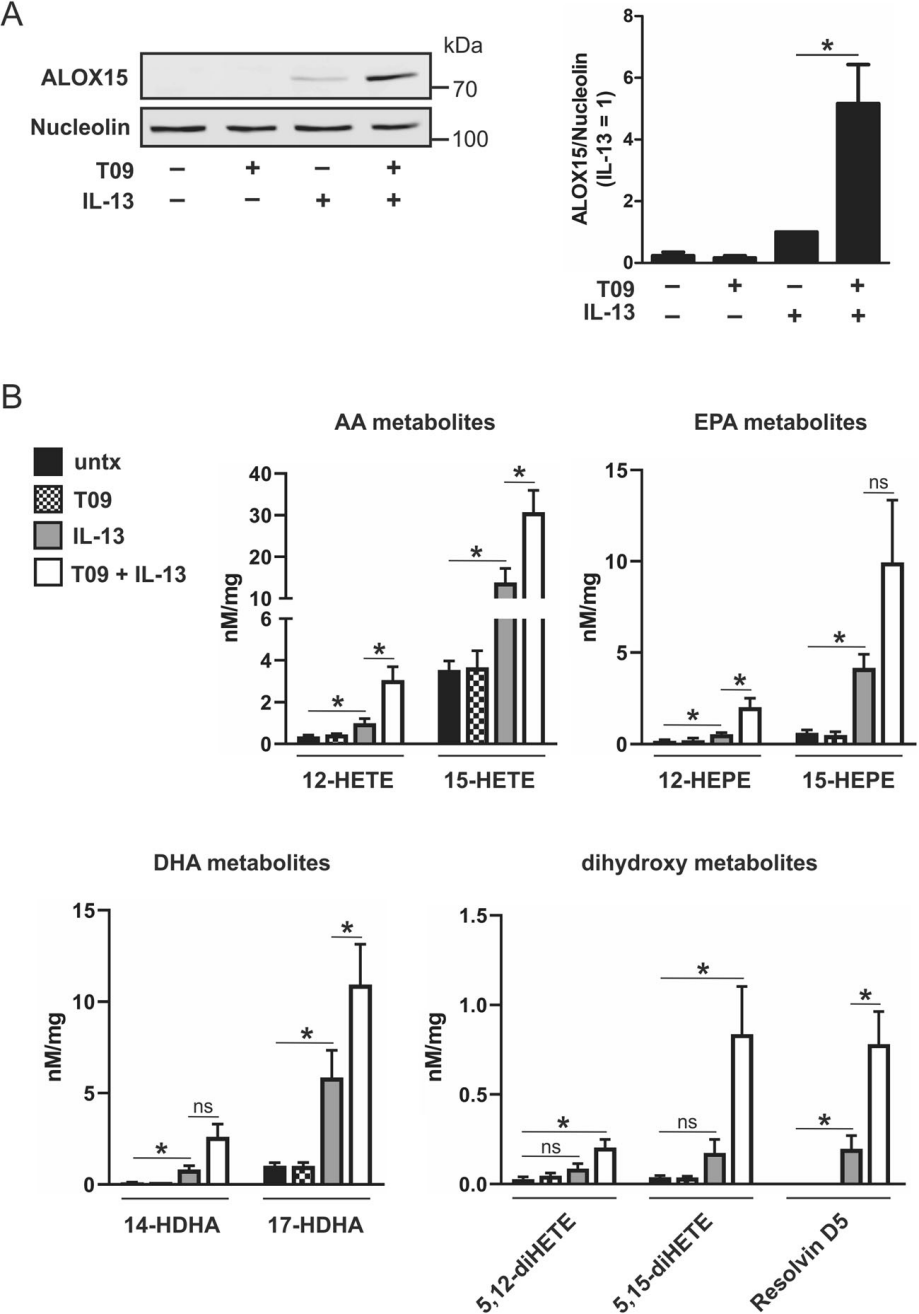
ALOX15 is modulated by LXR. **a** Representative western analysis of ALOX15 expression in macrophages pulsed with T09 for 3 h followed by stimulation with IL-13 (10 ng/ml) for 48 h (left panel), and corresponding densitometric analysis from three independent experiments (right panel); *P < 0.05 using one sample *t*-test. **b** Formation of AA-, EPA-, and DHA-derived lipid mediators in macrophages pulsed with T09 for 3 h followed by stimulation with IL-13 (10 ng/ml) for 48 h. Data are presented as mean ± SE from four independent experiments. Statistical analysis was performed using one-way ANOVA with Bonferroni post hoc means comparisons (*P < 0.05; ns = not significant).

Of the Th2 cytokine-dependent genes potentiated by LXR activation, both ALOX15 and IL1RN are linked to resolution of inflammation [29, 30]. Essentially absent in naive and pro-inflammatory macrophages, ALOX15 is highly expressed in proresolving macrophages where it facilitates the biosynthesis of SPMs [31]. To confirm enhanced protein expression of ALOX15 in cells primed with T09, we performed western analysis. Not detected in naive or T09-primed macrophages, ALOX15 protein was upregulated following stimulation with IL-13 and synergistically increased in T09-primed, IL-13-stimulated cells (Fig. 4a). To determine whether enhanced ALOX15 protein is capable of synthesizing increased amounts of lipid mediators, we performed a targeted lipidomic screen for SPMs and SPM precursors derived from PUFA substrates AA, DHA, and EPA by LC–MS/MS. AA-derived lipid precursors, 12-HETE and 15-HETE, EPA-derived lipid precursors, 12-HEPE and 15-HEPE, and DHA-derived lipid precursors, 14-HDHA and 17-HDHA, were all increased in IL-13-stimulated macrophages compared to naive or T09-primed cells, and were further increased in IL-13-stimulated cells primed with T09 (Fig. 4b). Furthermore, only LXR-primed, IL-13-stimulated macrophages synthesized significant levels of the dihydroxy metabolites 5,12-diHETE and 5,15-diHETE. The SPM resolvin D5 (RvD5) was increased in cells stimulated with IL-13 compared to naive macrophages, and its synthesis was further enhanced after T09-priming (Fig. 4b). As ALOX15 expression determines the capacity for temporal synthesis of SPMs and SPM precursors in AAMs [29], our data show priming cells, through LXR activation, enhances ALOX15 expression and activity.

### Engulfment of ACs potentiates IL-13-dependent ALOX15 expression

**Fig. 5:**
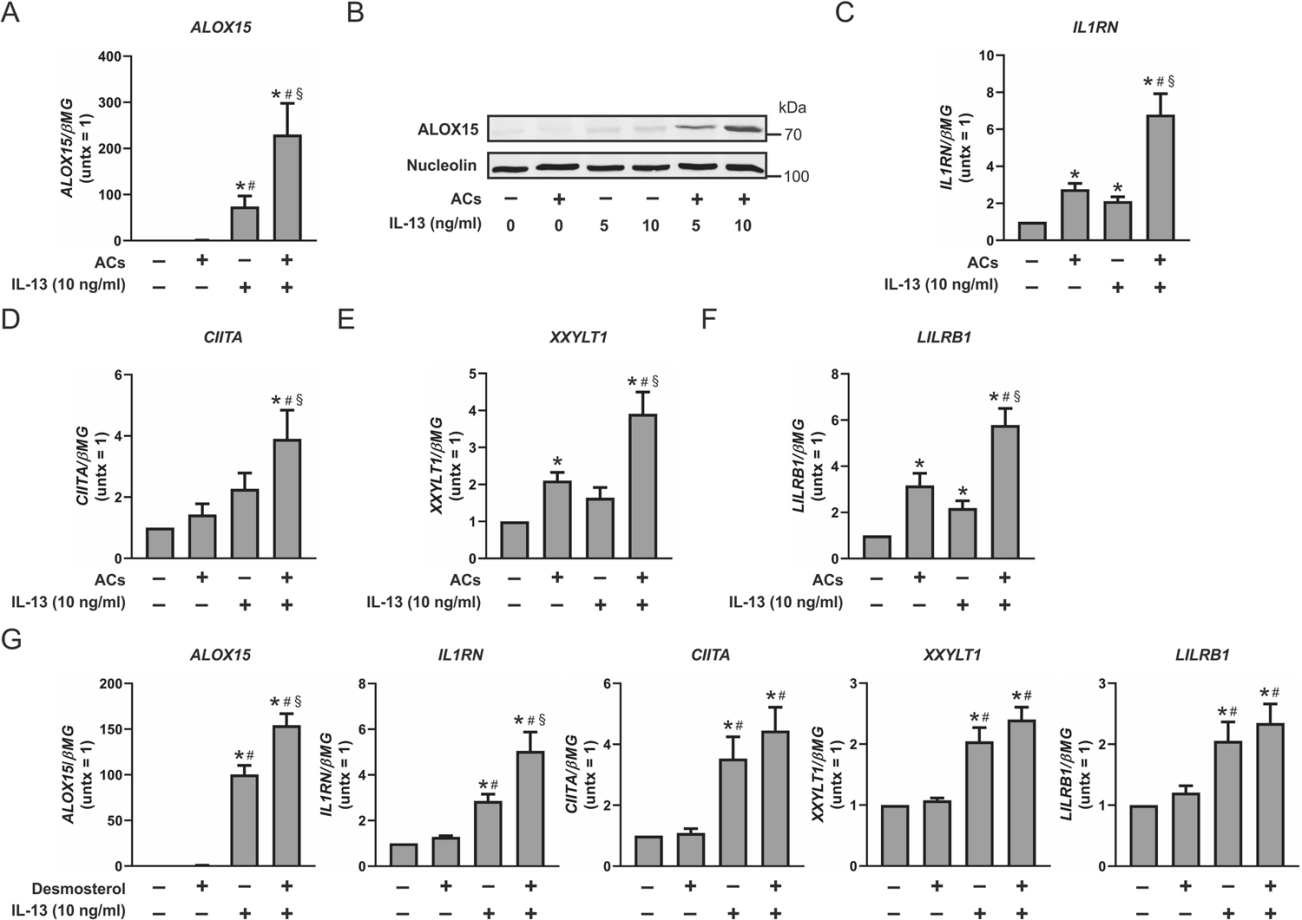
Engulfment of apoptotic cells potentiates IL-13-dependent ALOX15 expression. ALOX15 **a** gene expression in macrophages cocultured with ACs for 3 h followed by stimulation with IL-13 (10 ng/ml) for 24 h and **b** protein expression in macrophages cocultured with ACs for 3 h followed by stimulation with IL-13 (5 and 10 ng/ml) for 48 h. **c** IL1RN, **d** CIITA, **e** XXYLT1, and **f** LILRB1 gene expression in macrophages cocultured with ACs for 3 h followed by stimulation with IL-13 (10 ng/ml) for 24 h. **g** ALOX15, IL1RN, CIITA, XXYLT1, and LILRB1 gene expression in macrophages pretreated with desmosterol (10 µM) for 16 h then cotreated with desmosterol (10 µM) and IL-13 (10 ng/ml) for 24 h. Data in **a** and **c**–**g** are presented as mean ± SE from at least three independent experiments. Statistical analysis was performed using one-way ANOVA with Bonferroni post hoc means comparisons (*P < 0.05 vs untreated control; ^#^*P* < 0.05 vs ACs or desmosterol; ^§^*P* < 0.05 vs IL-13).

To determine whether efferocytosis-induced LXR activation recapitulates the potentiation of Th2-dependent genes ALOX15, IL1RN, CIITA, XXYLT1, and LILRB1 by T09-priming, we cocultured macrophages with apoptotic Jurkat cells as described in Fig. 1. ACs alone failed to induce ALOX15 mRNA. However, concomitant stimulation with ACs and IL-13 potentiated ALOX15 gene expression compared to IL-13 alone (Fig. 5a). Essentially absent in naive and T09-primed macrophages, ALOX15 protein was potentiated in a dose-dependent manner following concomitant stimulation of ACs and IL-13 compared to IL-13 alone (Fig. 5b). Although coculture with ACs alone upregulated gene expression of IL1RN, XXYLT1, and LILRB1 in macrophages, likely through LXR-independent mechanisms, concomitant stimulation of ACs and IL-13 potentiated the expression of IL1RN, CIITA, XXYLT1, and LILRB1 compared with IL-13 alone, thereby recapitulating the effects of T09-priming (Fig. 5c–f). Since sterol intermediates shown to activate LXR, including desmosterol and dihydrolanosterol [25, 26], were increased in efferocytic macrophages (Fig. 1e) and treatment with desmosterol alone enhanced LXR-dependent gene expression (Fig. 2a), we next determined whether desmosterol also recapitulates the potentiation of Th2-dependent genes. Macrophages pretreated with desmosterol followed by IL-13 stimulation exhibited increased gene expression of ALOX15 and IL1RN (Fig. 5g), albeit to a lesser extent than AAMs cocultured with ACs (Fig. 5a, c) or primed with T09 (Fig. 3f, g). Gene expression of CIITA, XXYLT1, and LILRB was modestly, but not significantly, increased in AAMs pretreated with desmosterol compared to IL-13 stimulation alone (Fig. 5g). These results confirm coordinated signaling by LXR agonists, both pharmacological or AC-derived, followed by Th2 cytokines potentiates the expression of ALOX15 and IL1RN in human macrophages but also suggest that AC-mediated potentiation of ALOX15, IL1RN, CIITA, XXYLT1, and LILRB1 in AAMs is likely not mediated exclusively through increased intracellular levels of desmosterol.

**Fig. 6:**
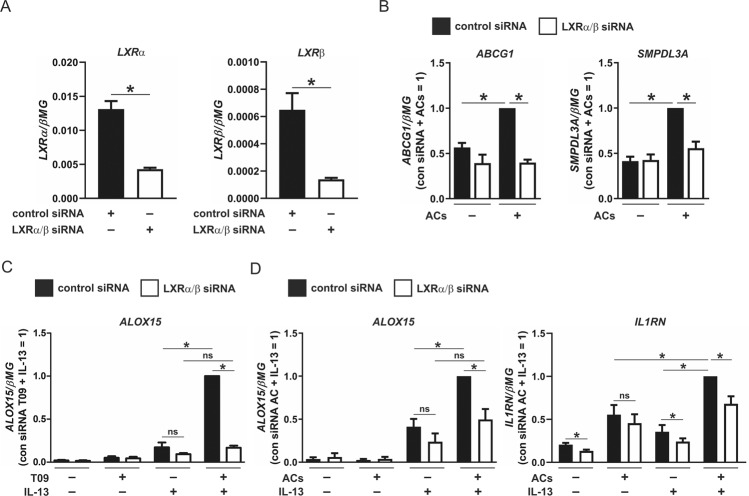
Gene expression in LXR knockdown macrophages. **a** Gene expression of LXRα and LXRβ in naive macrophages transfected with control or dual LXRα/β siRNAs. **b** Expression of LXR-dependent genes ABCG1 and SMPDL3A in control and dual LXRα/β knockdown macrophages cocultured with ACs for 3 h. **c** Gene expression of ALOX15 in control and dual LXRα/β knockdown macrophages pulsed with T09 (1 µM) for 3 h followed by 24-h stimulation with IL-13 (10 ng/ml). **d** Gene expression of ALOX15 and IL1RN in control and dual LXRα/β knockdown macrophages cocultured with ACs for 3 h followed by stimulation with IL-13 (10 ng/ml) for 24 h. Data are presented as mean ± SE from at least three independent experiments. Statistical analysis was performed with two-tailed Student’s *t* test (*P < 0.05) in **a**, one sample *t*-test (*P < 0.05) in **b**, and both two-tailed Student’s *t* test (*P < 0.05) and one sample *t*-test (*P < 0.05) in **c** and **d**. Statistical analysis was performed with one sample *t*-test (*P < 0.05), Student’s *t* test (^#^*P* < 0.05), and one-way ANOVA with Bonferroni post hoc means comparisons (^§^*P* < 0.05 vs untreated siRNA-transfected; ns = not significant).

To confirm the role of LXRs in the potentiation of ALOX15 by ACs, we used siRNAs to knockdown LXRs. As ACs promote induction of LXR-dependent gene expression through activating both LXRα and LXRβ in macrophages [9], we transiently transfected cells with control or siRNAs targeting both LXRα and LXRβ. Gene expression verified LXRα as the predominant LXR isoform in primary human macrophages and confirmed the efficiencies of the double knockdown (DKD) (Fig. 6a). Furthermore, expression of classic LXR-dependent genes ABCG1 and SMPDL3A increased in control siRNA-transfected macrophages following coculture with ACs but not in LXRα/β DKD macrophages (Fig. 6b). With respect to ALOX15, its gene expression was significantly potentiated by concomitant stimulation of T09 and IL-13 compared with IL-13 alone in control siRNA-transfected macrophages but not in LXRα/β DKD cells (Fig. 6c). Although not to the same extent, ALOX15 gene expression was again enhanced by concomitant stimulation of ACs and IL-13 compared with IL-13 alone in control siRNA-transfected macrophages but was attenuated in LXRα/β DKD cells (Fig. 6d). Similarly, IL1RN gene expression, which was increased by ACs in both control siRNA-transfected and LXRα/β DKD cells, was also enhanced by concomitant stimulation of ACs and IL-13 compared with IL-13 alone in control siRNA-transfected macrophages but was also attenuated in LXRα/β DKD cells (Fig. 6d). To understand how LXRs modulate ALOX15 expression, we used the in silico online motif discovery tools HOMER and TRANSFAC to identify putative LXREs containing the consensus DR4 motif nearby the ALOX15 gene (Supplementary Fig. 2A) [32]. Although we identified several putative LXRα binding regions, we were unable to detect enhanced LXRα binding following stimulation with T09 or concomitant stimulation with T09 and IL-13 using chromatin immunoprecipitation (ChIP) (Supplementary Fig. 2B). Previous experiments performed in macrophages have shown LXRs bind a large number of sites with no DR4 motif resemblance as well as to DR1 elements within PPAR-specific PPREs [32, 33]. Also considering data from recent ChIP-seq experiments showing LXRα and LXRβ occupy both overlapping and exclusive genomic regulatory sites [34], a more extensive profiling of LXRα and LXRβ binding is necessary to fully elucidate LXR-mediated regulation of ALOX15.

### Sterol intermediates in ACs

**Fig. 7:**
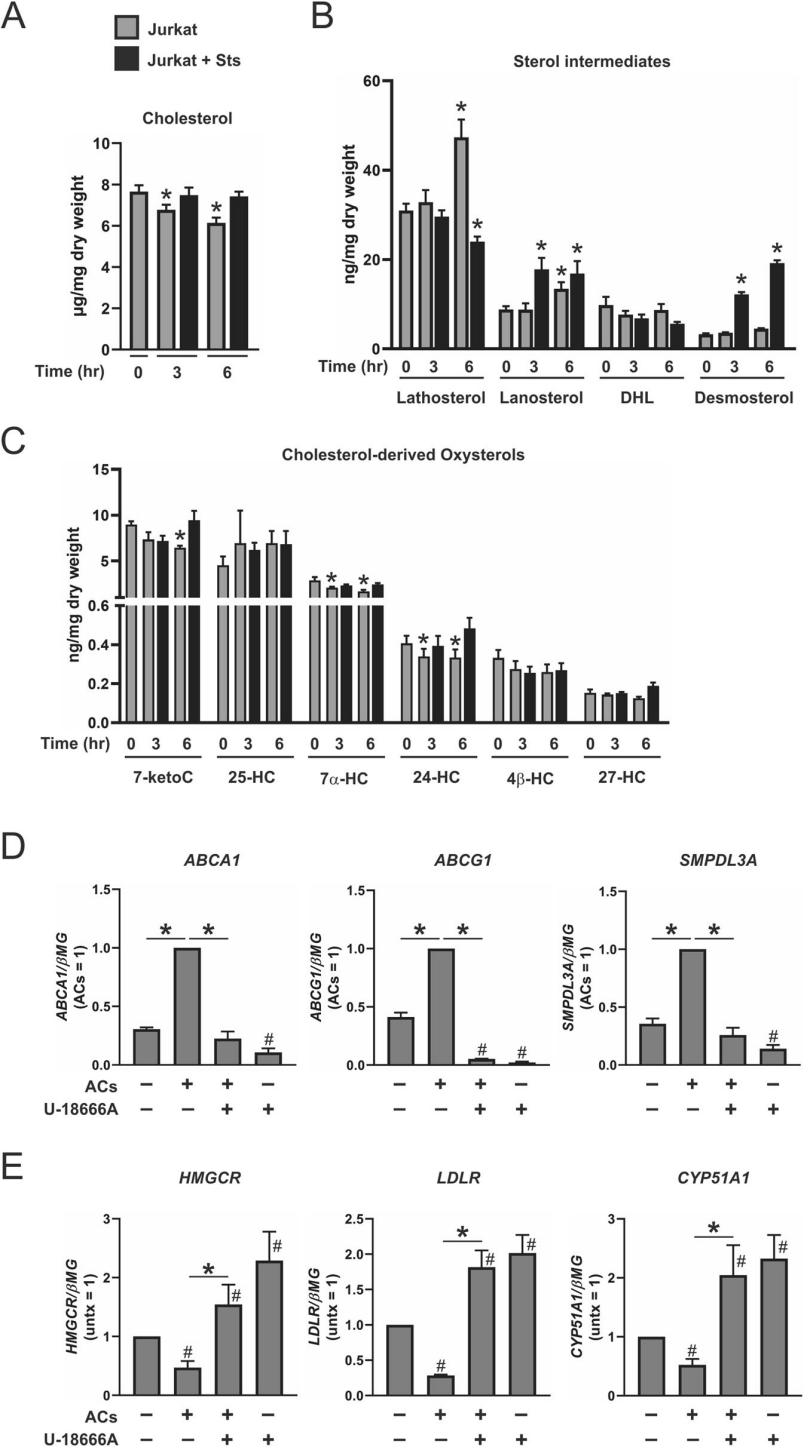
Sterols in apoptotic cells. **a** Total cholesterol, **b** sterol intermediates lathosterol lanosterol, DHL (dihydrolanosterol), and desmosterol, and **c** cholesterol-derived oxysterols in Jurkat cells treated with or without staurosporine (0.5 µM) in serum-free media for 0, 3, and 6 h (7-ketoC, 7-ketocholesterol; 25-HC, 25-hydroxycholesterol; 7α-HC, 7alpha-hydroxycholesterol; 24-HC, 24-hydroxycholesterol; 4β-HC, 4beta-hydroxycholesterol; 27-HC, 27-hydroxycholesterol). Expression of **d** LXR-dependent genes ABCA1, ABCG1, and SMPDL3A, and **e** SREBP-2-dependent genes HMGCR, LDLR, and CYP51A1 in human monocyte-derived naive macrophages pretreated with U-18666A (5 µM) for 1 h then cotreated with ACs for 3 h. Data are presented as mean ± SE from at least four independent experiments. Statistical analysis was performed using one-way ANOVA with Bonferroni post hoc means comparisons in **a**–**c** (*P < 0.05 vs time 0). For **d** and **e**, statistical analysis was performed with two-tailed Student’s *t* test (**d** ^#^*P* < 0.05 vs untreated control; **e** *P < 0.05 vs ACs) and one sample *t*-test (**d** *P < 0.05 vs ACs and **e** ^#^*P* < 0.05 vs untreated control).

Our findings indicate LXRs link AC engulfment to enhanced ALOX15 and IL1RN gene expression; however, the mechanism underlying the accumulation of sterol intermediates in efferocytic macrophages is not clear. Previous studies, including those by Brown and Goldstein, reported sterol-regulated genes are activated at an early stage in the apoptotic cascade [35, 36], even preceding the externalization of phosphatidylserine on the plasma membrane [37]. To determine whether accumulated sterol intermediates in efferocytic macrophages are derived from ACs, we performed lipidomic analysis of apoptotic Jurkat cells. Since apoptosis was induced with staurosporine in media free of serum, we also measured sterols of untreated, viable Jurkat cells at corresponding time points to control for changes in cholesterol biosynthesis induced by serum removal. Total cholesterol levels did not change in ACs but were reduced in viable cells following three and 6-h serum withdrawal (Fig. 7a). Lanosterol and desmosterol levels increased 3 and 6 h following treatment with staurosporine, while lathosterol was reduced after six hours (Fig. 7b). Serum withdrawal increased levels of lathosterol and lanosterol after six hours but did not change levels of desmosterol in viable cells. Neither staurosporine nor serum withdrawal increased cholesterol-derived oxysterol levels, however viable cells contained less 7-ketocholesterol at six hours, as well as 7α-HC and 24-HC at both three and six hours (Fig. 7c). Following recognition and entrapment of ACs, cell corpses are targeted to lysosomes for digestion [11]. After digestion, AC-derived sterols are removed from lysosomes for subsequent intracellular trafficking via the coordinated action of Niemann–Pick type C (NPC) 1 (NPC1) and NPC2 proteins [38]. To confirm upregulation of LXR-dependent genes in efferocytic macrophages is mediated through exogenous AC-derived sterols, we blocked sterol transport from lysosomes by inhibiting NPC1 with the cationic amphiphile U-18666A [39]. Our results show gene expression of ABCA1, ABCG1, and SMPDL3A was increased following coculture with ACs but was completely ablated in cells treated with U-18666A (Fig. 7d), while gene expression of HMGCR, LDLR, and CYP51A1 was reduced following coculture with ACs but was rescued and even enhanced in cells treated with U-18666A (Fig. 7e).

## Discussion

Given the critical role of LXR in macrophage efferocytosis, we questioned whether LXR functions as a signaling node linking engulfment of ACs to enhanced Th2 cytokine-dependent gene expression. Thus, we used an in vitro coculture model of apoptotic Jurkat cells and primary human monocyte-derived macrophages to examine LXR signaling in efferocytic macrophages. Our findings showed efferocytic macrophages accumulated the sterol intermediates lathosterol, desmosterol, lanosterol, and dihydrolanosterol and upregulated LXR-dependent gene expression including ABCA1, ABCG1, SMPDL3A, and MERTK, but simultaneously suppressed the expression of SREBP-2 target genes HMGCR, LDLR, CYP51A1, and DHCR24.

Our findings that reciprocal modulation of LXR- and SREBP-2-dependent gene expression coincide with increased sterol intermediates of the Kandutsch–Russell and Bloch pathways are in line with previous reports showing accumulation of sterol intermediates coordinately induce LXR and repress SREBP-2 target genes [15, 24, 25]. Although our lipidomic analysis determined four sterol intermediates, we cannot overlook the possibility of the accumulation of additional intermediates with LXR and SREBP-2 modulating properties. Not all sterol intermediates demonstrate dual and reciprocal regulation of LXR and SREBP-2. Whereas 24,25-dihydrolanosterol, FF-MAS, and desmosterol both activate LXR and inhibit SREBP-2 [15, 24, 40], zymosterol only activates LXR without inhibiting SREBP-2 [25], while lanosterol neither activates LXR nor inhibits SREBP-2 [24, 25, 40]. The contribution of additional sterol intermediates including dihydro-FF-MAS, T-MAS, dihydro-T-MAS, and zymostenol to LXR and SREBP-2 regulation remain unclear. Desmosterol directly binds LXRα and LXRβ and induces the recruitment of the cofactor SRC-1 [25]. In addition to its accumulation in efferocytic macrophages, desmosterol is the most abundant endogenous LXR activator in murine macrophage foam cells as well as human atherosclerotic plaques [41]. Our observations that LXR- and SREBP-2-dependent gene expression is coordinately and reciprocally regulated is also consistent with gene expression in macrophages cocultured with apoptotic intestinal epithelial cells [42], apoptotic thymocytes [9], and apoptotic neutrophils [43] suggesting the reciprocal regulation of LXR and SREBP-2 transcriptional programs in efferocytic macrophages is likely a conserved response, irrespective of the AC-type.

After confirming LXR-dependent upregulation of gene expression in efferocytic macrophages, we used global transcriptome analyses to identify Th2 cytokine-dependent genes potentiated by LXR. Activating LXR with T09 potentiated the expression of Th2 cytokine-dependent genes ALOX15, IL1RN, CIITA, XXYLT1, and LILRB1. Although our manuscript focuses primarily on ALOX15 and to a lesser extent IL1RN, understanding how enhanced CIITA, LILRB1, and XXYLT1 expression impacts AAM function is an intriguing question considering that CIITA is critical for the expression of MHC class II genes and is thus a key regulator of the adaptive immune response [44], while the immunomodulatory receptor LILRB1 has been shown to repress macrophage effector function upon interaction with MHC class I molecules [45]. In contrast to ALOX15 and IL1RN expression, CIITA, XXYLT1, and LILRB1 expression was potentiated in AAMs cocultured with ACs or primed with T09, but not in AAMs pretreated with exogenous desmosterol. This discrepancy highlights the disparity in transcriptional responsiveness of particular genes to endogenous and synthetic regulators of LXRs, as recently reported by Muse et al. [15] and also suggests efferocytosis-mediated LXR activation is unlikely mediated exclusively by increasing desmosterol. Our findings also accentuate the contribution of LXR-independent signaling in efferocytic macrophages as expression of IL1RN, LILRB1, and XXYLT1 is seen in cells cocultured with ACs alone but not in macrophages primed with T09 or treated with desmosterol. This is not surprising considering the reported contribution of various nuclear receptors in efferocytosis including PPARƴ [13], which modulates IL1RN expression in THP-1 cells [46]. Our global transcriptome analyses also indicated that the expression of common Th2 cytokine-dependent genes associated with the AAM phenotype including MRC1, TGM2, CD209, ALDH1A2, and MAOA were not potentiated by LXR priming. Gene expression of several Th2 cytokine-dependent chemokines was unaltered and even suppressed in LXR-primed macrophages mirroring the reported suppression of LPS-induced chemokines in LXR-primed murine macrophages [47].

Identification of ALOX15 as a Th2 cytokine-dependent gene uniquely potentiated by LXR agonists is in consonance with its established role in resolving inflammation and coordinating AC clearance. Mediating the stereoselective oxygenation of free and esterified PUFAs, ALOX15 is indispensable for the synthesis of SPMs [48]. Recent reports in human AAMs showed ALOX15 protein levels directly determine the temporal synthesis of SPMs [29], which strongly supports our lipidomic results showing the synthesis of ALOX15-mediated lipid mediators, including RvD5, correspond with ALOX15 protein levels in macrophages. With respect to ALOX15 substrates, LXR activation both increases levels of long-chain PUFAs DHA and AA [49], and modulates membrane phospholipid composition by incorporating PUFAs at the *sn-*2 site of lysophospholipids through enhanced expression of LPCAT3 [50]. Additional evidence linking LXR to ALOX15 was recently reported by Körner et al. who demonstrated inhibition of DHCR24 led to an anti-inflammatory/proresolving phenotype in a murine peritonitis model. Specifically, DHCR24 inhibition increased desmosterol in peritoneal lavage cells which expressed higher levels of Alox15 mRNA and synthesized increased amounts of ALOX15-derived 15-HETE and 17-HDHA [28].

**Fig. 8:**
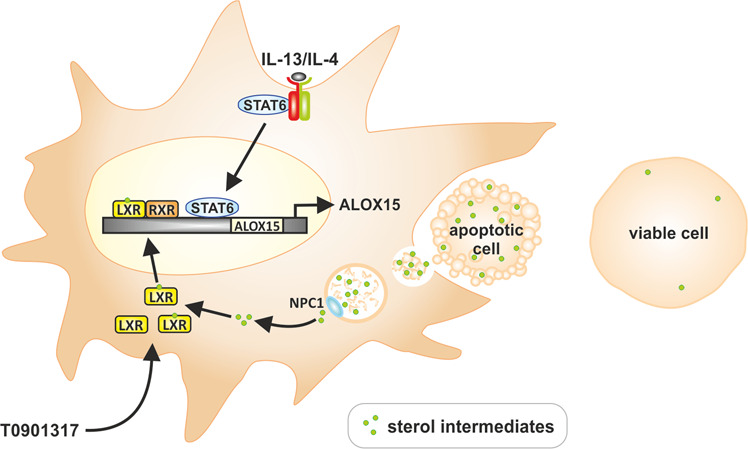
Mechanistic scenario for induction of ALOX15. Accumulation of sterol intermediates derived from ACs, or synthetic LXR ligand T0901317, activate LXR to facilitate cellular sterol metabolism while simultaneously priming macrophages for enhanced expression of Th2 cytokine-dependent gene ALOX15.

ALOX15 is also strongly implicated in the clearance of ACs. Although dispensable for their direct uptake, the induction of ALOX15 protein appears to be a consequence of saturated-efferocytosis at the cellular level [51, 52]. In this regard, ALOX15 functions to orchestrate and allocate the cellular clearance of ACs through generating oxidation products of phosphatidylethanolamine which block the uptake of ACs by inflammatory monocytes [53]. In peritoneal macrophages, ALOX15 protein was upregulated only in cells that engulfed a high number of ACs [52], implying accumulation of apoptotic debris facilitates ALOX15 expression. Since LXR plays a major role in integrating AC-derived sterols, our findings support a mechanistic scenario in which accumulation of sterol intermediates derived from ACs activate LXR to facilitate cellular sterol metabolism while simultaneously priming macrophages for enhanced expression of Th2 cytokine-dependent genes ALOX15 and IL1RN (Fig. 8).

To determine whether accumulated sterol intermediates in efferocytic macrophages might be derived from ACs we performed lipidomic analysis of apoptotic Jurkat cells. We noticed increased Bloch pathway intermediates, desmosterol and lanosterol, but reduced Kandutsch–Russell intermediate lathosterol, suggesting a potential alteration in the enzymatic activity of DHCR24 which regulates flux through the two pathways, and whose inhibition culminates in the accumulation of desmosterol [54]. Although western analysis revealed unchanged DHCR24 protein levels in apoptotic Jurkat cells (Supplementary Fig. 3), enzyme activity is regulated through post-translational modifications as well as by changes in subcellular localization [55, 56]. Given that sterol-regulated genes are activated at early stages in the apoptotic cascade [37], alterations in DHCR24 activity should be explored.

Unresolved inflammation contributes to the development of several pathological conditions including insulin resistance, atherosclerosis, inflammatory bowel disease, and rheumatoid arthritis [57]. Although traditional therapies targeting chronic inflammatory disease aim to suppress effector pathways, stimulating the resolution process is a promising new approach to reversing inflammatory conditions without curtailing the overall inflammatory response. As ALOX15 protein levels determine the cellular capacity for SPM synthesis, manipulating macrophage ALOX15 expression with LXR modulators may provide new possibilities to resolve chronic inflammatory disease.

## Supplementary information

Supplementary Method

Supplementary Figure S1

Supplementary Figure S2

Supplementary Figure S3

Supplementary Table 1

Supplementary Table 2

Supplementary Table 3

Supplementary Table 4

Supplementary Table 5
