# Supplementary material for: Efferocytosis potentiates the expression of arachidonate 15-lipoxygenase (ALOX15) in alternatively activated human macrophages through LXR activation
Source: Cell Death Differ. 2020 Nov 11;28(4):1301–16. doi: 10.1038/s41418-020-00652-4 (PMC8027700; doi:10.1038/s41418-020-00652-4)
Supplement: Supplementary file 1 — Supplementary Method [file 41418_2020_652_MOESM1_ESM.docx]

Chromatin Immunoprecipitation (ChIP)

Human primary macrophages were fixed in 1% paraformaldehyde, quenched with 0.125 M glycine, and washed in PBS. Cells were lysed (10 mM HEPES/KOH pH 7.9, 1 mM EDTA, 85 mM KCl, 0.5% NP-40) to release cytosolic proteins and debris and the nuclear pellet was then lysed in nuclei lysis buffer (50 mM Tris–HCl pH 7.4, 10 mM EDTA, 1% SDS, 0.5% Empigen BB). Extracts were sonicated with 425-600 µm glass beads (Sigma) using a Branson Sonifier until DNA fragments were 300-500 bp in average size as assessed by agarose gel electrophoresis. Soluble chromatin was diluted with dilution buffer (0.5% Triton X-100, 2 mM EDTA, 20 mM Tris–HCl pH 7.4, 100 mM NaCl) and pre-cleared with sepharose CL-4B beads (Sigma-Aldrich) for 2 h at 4°C. Chromatin was incubated with 5 µg LXRα (Active Motif; catalog # 61175) or IgG (Abcam; catalog # ab171870) antibodies overnight at 4°C then captured with Protein A/G beads (Santa Cruz Biotechnology). Antibody/bead complexes were washed once with low salt buffer (0.1% SDS, 1% Triton X-100, 2 mM EDTA, 20 mM Tris–HCl pH 7.4, 150 mM NaCl), once with high salt buffer (0.1% SDS, 1% Triton X-100, 2 mM EDTA, 20 mM Tris–HCl pH 7.4, 500 mM NaCl) and twice with LiCl buffer (250 mM LiCl, 10 mM Tris–HCl, pH 7.4, 1% NP-40, 1% sodium deoxycholate, 1 mM EDTA). The beads were eluted in elution buffer (100 mM NaHCO3, 1% SDS), reverse crosslinked with proteinase K overnight, digested with RNAse for 2 h at 37°C, then purified using Qiagen Ampure purification kit (Qiagen) and again eluted in 100 μl of elution buffer. Input and recovered DNA were analyzed using quantitative PCR using the following primers corresponding to the region encompassing the LXRE of the ABCA1 promoter ((5’-TGCCGGGACTAGTTCCTTTTA-3’ (forward) and 5’-CATAAACAGAGGCCGGGAAC-3’ (reverse)) as well as regions encompassing potential LXRα binding sequences in the vicinity of ALOX15 ((primer pair 1: 5’-CATGCCCGGCTACTTTTTGT-3’ (forward) and 5’-GCAGGTGGATCACTTGAGGT-3’ (reverse); primer pair 2: 5’-TTTCAGTCGAGATGGGGTTTCA-3’ (forward) and 5’-ACATGATGGCTCATGGCTGTAA-3’ (reverse); primer pair 3: 5’-TTCAGTCGAGATGGGGTTTCAC-3’ (forward) and 5’-TAAACAATTAAGCAGGGCCGGA-3’ (reverse); primer pair 4: 5’-CCAATCAACTCCGCCTACCG-3’ (forward) and 5’-CCCTCCCGTCAAGATAGTGG-3’ (reverse); primer pair 5: 5‘-TTCAGCCCCAGTCCAAAAGG-3’ (forward) and 5’-TGCTGTACCAGGCGTTGATT-3’ (reverse); primer pair 6: 5’-CGCCTGTAATCCCAACACTTTG-3’ (forward) and 5’-GAGGTTTTGCCATGTTGGTCAG-3’ (reverse); primer pair 7: 5’-CTCACGCCTGTAATCCCAACA-3’ (forward) and 5’-GGTTTTGCCATGTTGGTCAGG-3’ (reverse); primer pair 8: 5’-ATAATCCCAGCAGTTTGGGAGG-3’ (forward) and 5’-AGGGTTTCACCATGTTGTCCAG-3’ (reverse); and primer pair 9: 5’-TGGCTCATGTCTATAATCCCAGC-3’ (forward) and 5’-TTTCACCATGTTGTCCAGGCT-3’ (reverse)). Data are presented as ChIP-quantitative PCR fold enrichment of LXRα ChIP over input DNA relative to IgG negative control.
