# Supplementary figures and images for: Efferocytosis potentiates the expression of arachidonate 15-lipoxygenase (ALOX15) in alternatively activated human macrophages through LXR activation
Source: Cell Death Differ. 2020 Nov 11;28(4):1301–16. doi: 10.1038/s41418-020-00652-4 (PMC8027700; doi:10.1038/s41418-020-00652-4)

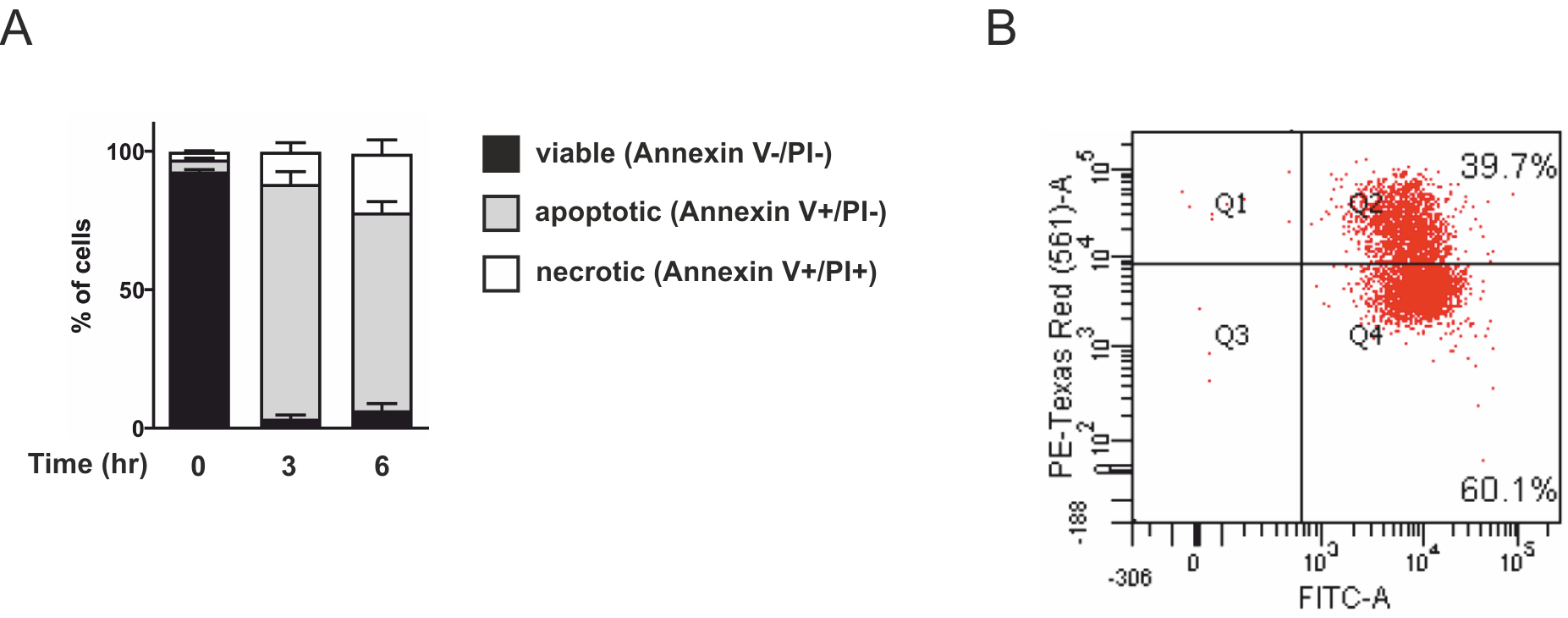

Supplement: Supplementary file 2 — Supplementary Figure S1 [file 41418_2020_652_MOESM2_ESM.png]

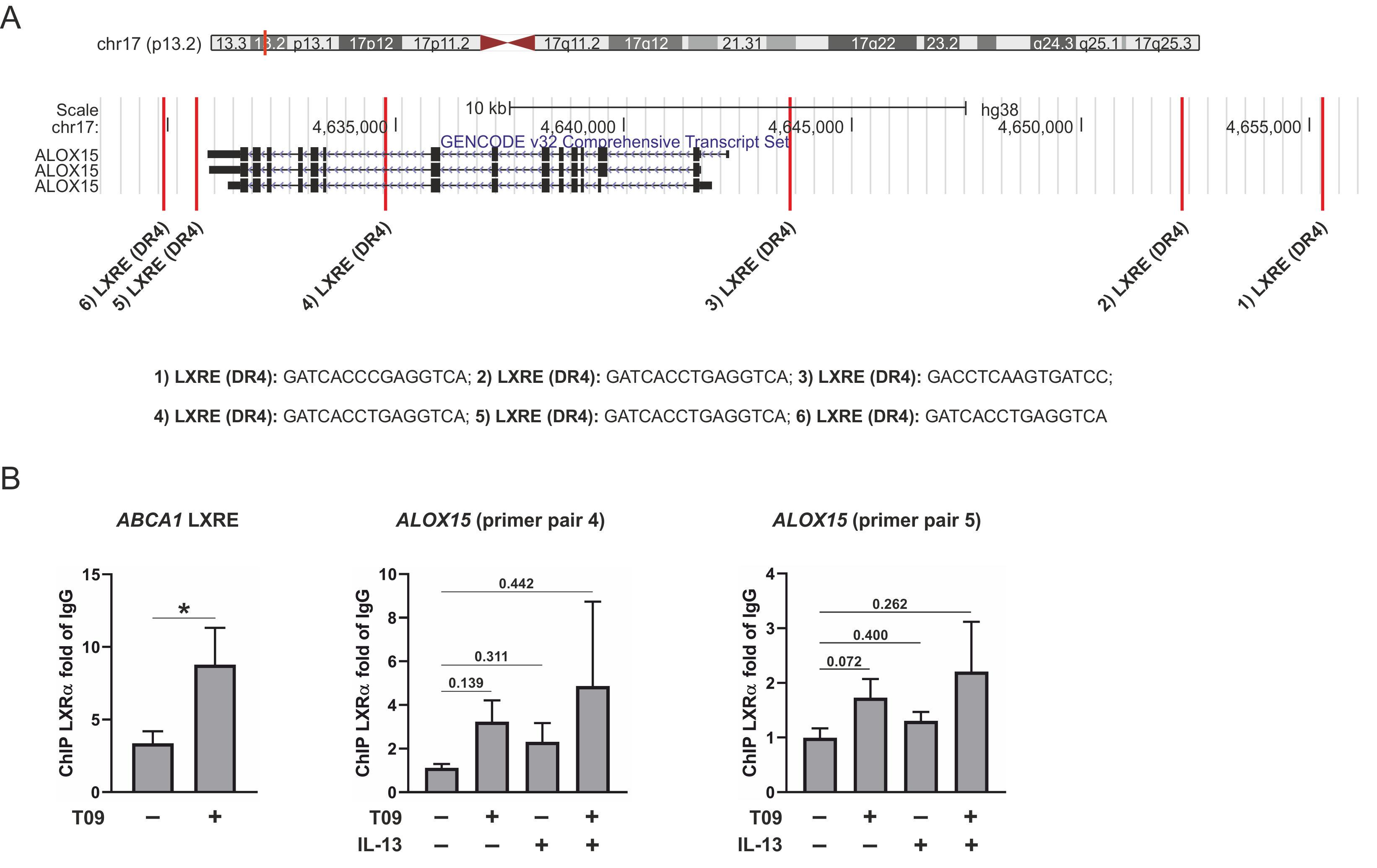

Supplement: Supplementary file 3 — Supplementary Figure S2 [file 41418_2020_652_MOESM3_ESM.png]

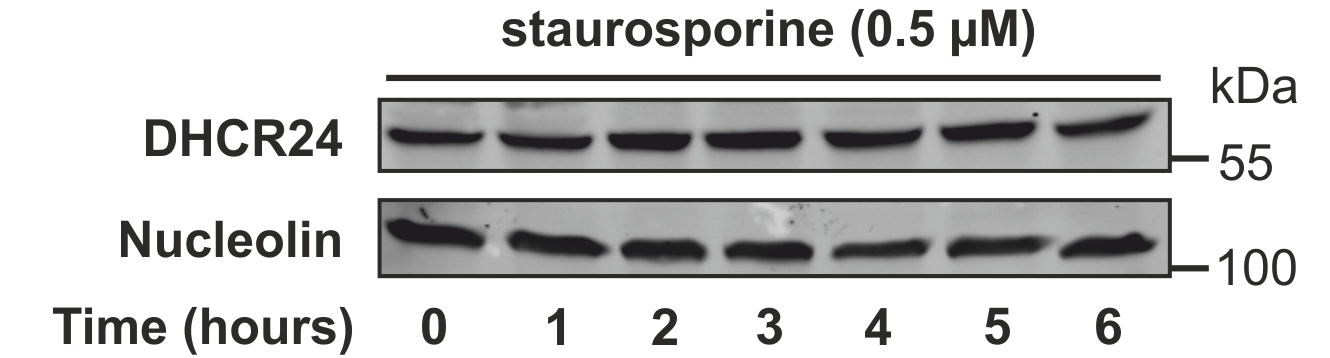

Supplement: Supplementary file 4 — Supplementary Figure S3 [file 41418_2020_652_MOESM4_ESM.png]
